# Foodborne Botulism, I Only Had Nacho Cheese: A Case Report

**DOI:** 10.7759/cureus.1666

**Published:** 2017-09-08

**Authors:** Saad A Choudhry, Muhammad Jahanzaib Anwar, Muhammad Afzal, Abdul Shah

**Affiliations:** 1 Internal Medicine, University of California Davis; 2 Department of Internal Medicine, Rush University Medical Center; 3 Pulmonary and Critical Care, Sutter Medical Center Sacramento; 4 Intermountain Healthcare

**Keywords:** foodborne, botulism, clostridium botulinum, home canned food, botulinum antitoxin, nacho cheese

## Abstract

A 32-year-old female presented to the emergency department with complaints of diplopia, followed by dyspnea, chest tightness, congestion, and dysphagia. The patient was resuscitated and initial investigations were done. Within a few hours of the admission, she started developing signs of respiratory failure and was intubated and placed on the mechanical ventilator. The patient denied any ingestion of exotic food, shellfish, raw meat, or raw fish. The patient also denied traveling to any exotic place or recent camping trips. The edrophonium tensilon test and lumbar puncture came out to be negative. The botulinum toxin test was positive, the patient was started on botulinum antitoxin, and the rest of symptomatic treatment was continued. The Centre for Disease Control (CDC) tracked the events related to the presentation and found she had eaten nacho cheese from a gas station the day before the appearance of the symptoms. A total of 10 cases were associated with this source within days and one death was reported.

## Introduction

Foodborne botulism has been reported since as early as the eighteenth century when some ancient case reports described patients with a combination of dilated pupils and fatal muscle paralysis. The eighteenth century ended with some well-documented cases of outbreaks of “sausage poisoning” in Southern Germany that led to the need for early systematic botulinum toxin research. The first complete report with a complete description of the symptoms of foodborne botulism was reported between 1817 and 1822 by a German poet and district medical officer, Justin Kerner. At that time, he called it the “sausage poison” or the “fatty poison.” After 80 years of Kerner's work, in 1895, an outbreak of botulism in a Belgian town led to the discovery of the causative organism, Clostridium botulinum, by a professor of bacteriology at the University of Ghent―Dr. Emile Pierre van Ermengem. The Latin word for sausage is botulus, naming the bacterium as botulinum [1].

Recent studies and clinical experiences show that the most common source of foodborne botulism is often the canning of foods at home, which has a low acid content, such as green beans, corn, and beets. A common source of the illness in cold climate areas is fermented seafood. However, the use of oil-infused garlic, chili peppers, and baked potatoes, especially the foil-wrapped ones, are also found to be associated with the disease [2-3].

## Case presentation

A 32-year-old female presented to the emergency department with a few hours' history of shortness of breath and weakness associated with chest tightness, congestion, hoarseness of voice, and difficulty swallowing. The patient had presented 24 hours earlier with the complaint of diplopia for one day. The patient denied any ingestion of exotic food, shellfish, raw meat, raw fish, or other foods generally associated with botulism. The patient also denied traveling to any exotic place or recent camping trips. The general workup, including biochemical and hematological investigations, came out to be normal except for a mildly decreased serum calcium (7.9 mg/dl). The magnetic resonance imaging (MRI) scan of the brain was also normal. That day, she was discharged with an outpatient consultation with neurology, but she continued to have persistent diplopia. After a few hours of admission, her breathing started to get worse, she was intubated, placed on mechanical ventilation, and was admitted to the intensive care unit (ICU). On examination, she had a symmetrical weakness in all four limbs, with more in the upper limbs compared to the lower limbs. The tensilon test was performed, which was negative. A lumbar puncture was performed, which also came out to be normal. Blood was sent to be tested for botulinum toxin. In the meantime, symptomatic treatment was started and disease control authorities were involved. Her weakness progressively increased and while she was being treated, another case arrived at the hospital with a very similar presentation. Four days later, the botulinum toxin test came positive and the patient was started on botulinum antitoxin and the rest of symptomatic treatment was continued. The Centre for Disease Control (CDC) tracked the events related to both these patients and found out that they both had eaten nacho cheese from a gas station the day before the appearance of their symptoms. A total of 10 cases were associated with this source within days and one death was reported.

## Discussion

Botulism is a rare, neuroparalytic, potentially life-threatening disease caused by toxins released by a gram-positive spore-forming bacteria, the Clostridium botulinum. The toxin blocks acetylcholine receptors at neuromuscular junctions, which result in a descending type of flaccid paralysis of voluntary muscles. The first symptoms to appear are ptosis, diplopia, and dysarthria, eventually leading to full-body paralysis, including of the respiratory muscles. While the affected patients are fully alert and their sensory system is intact throughout the period of illness, the disease is lethal due to the involvement of respiratory muscles, which might lead to respiratory failure and death [4]. Recovery usually takes weeks to months. The patients should be admitted to the ICU and the availability of mechanical ventilation should be ensured promptly if needed. The antitoxin should be administered early in the disease to decrease the natural course of the disease. The prognosis depends on early diagnosis and administration of the antitoxin, which may reduce the further deterioration of muscle weakness and the severity of the disease [5].

Foodborne botulism occurs when food containing preformed toxin is ingested. Clostridium botulinum spores are present in the environment [6] but the growth and development of the toxins only occur under specific conditions such as an anaerobic, low salt, and low acid environment. “Bacterial growth is inhibited by refrigeration below 4°C, heating above 121°C, high water activity, or acidity (pH <4.5)” [7]. “The toxin is destroyed by heating to 85°C for at least five minutes and spores are inactivated by heating to 121°C under pressure of 15–20 lb/in^2^ for at least 20 minutes” [7].

Improper food handling practices are the most important cause of foodborne botulism in the United States as well as the rest of the world. Between 1990 and 2000, 160 cases of foodborne botulism were reported, in which 263 people were affected. According to the available data, eight deaths were recorded. Out of the 160 events, 58 occurred in Alaska, affecting 103 people. In most of these cases, the identified food source was fermented aquatic mammal tissue, beaver tails, seal flippers, and seal oil. Another source identified was fish and fish products such as fermented salmon head, white fish, and fish eggs [3].

In the rest of the states, including Hawaii, 102 events occurred, affecting 160 people. The food sources identified were diverse, including homemade canned foods, commercial foods, as well as restaurant-prepared foods [3]. But the reason remained―improper storage and preservation like inadequate refrigeration, use of sealed plastic bags and cans, and their exposure to sunlight and inability to heat the foods to a temperature that might kill the toxin. In this time period, there were 37 cases in which no identifiable food source could be attributed to the disease [3].

According to the CDC reports between 2001 and 2015, a total of 278 cases of foodborne botulism and 23 deaths were reported. One of the biggest outbreaks occurred in Ohio in 2015, in which 27 cases were reported and the food was confirmed to be potato salad/macaroni. Other outbreaks were caused by different home-canned foods like chili sauce, prune, beans, mushrooms, and other home-canned vegetables. During this time, there were 45 cases that could not be attributed to a specific food source [3].

## Conclusions

Foodborne botulism, while rare, remains a public health emergency in the United States. Due to advances in medicine, it has become treatable and manageable but still poses a huge health problem due to its severity and epidemic potential. Botulism should always be included in the differential diagnosis of a patient with similar signs and symptoms, as early diagnosis could be critical in the management and, eventually, the prognosis of an affected patient. While taking the history of the patient, all foods should be taken into account just before the development of the symptoms rather than asking about the food sources generally associated with the disease. Even when there is no history of intake of foods associated with botulism, it should remain in the differentials until proven otherwise. Home-canned foods and Alaska native foods remain the leading cause of botulism in the United States. Restaurant-associated outbreaks continue to account for numerous illnesses. There should be a tight check over the packaging of known foods that have the potential to thrive Clostridium botulinum, and all such facilities should be federally regulated. All suspected cases of botulism should be reported to public health authorities immediately. Prompt epidemiologic investigation helps prevent additional cases and can identify new risk factors for intoxication.
